# Enhanced Rheological and Structural Properties of the Exopolysaccharide from *Rhizobium leguminosarum* VF39 Through NTG Mutagenesis

**DOI:** 10.3390/polym16223179

**Published:** 2024-11-15

**Authors:** Kyungho Kim, Eunkyung Oh, Sohyun Park, Jae-pil Jeong, Sobin Jeon, Sujin Lee, Younghyun Shin, Seunho Jung

**Affiliations:** 1 Department of Bioscience and Biotechnology, Microbial Carbohydrate Resource Bank (MCRB), Konkuk University, 120 Neungdong-ro, Gwangjin-gu, Seoul 05029, Republic of Korea; rudgh971225@naver.com (K.K.); eunkyung_5@naver.com (E.O.); so63991@naver.com (S.P.); jjp0531@naver.com (J.-p.J.); syh4969@naver.com (Y.S.); 2 Department of System Biotechnology, Microbial Carbohydrate Resource Bank (MCRB), Konkuk University, 120 Neungdong-ro, Gwangjin-gu, Seoul 05029, Republic of Korea; wjsthqls030414@naver.com (S.J.); 24mnb@naver.com (S.L.)

**Keywords:** exopolysaccharides (EPSs), NTG mutagenesis, viscoelasticity, thermal stability, biocompatibility, FT-IR, NMR

## Abstract

Microbial exopolysaccharides (EPSs) are biopolymer materials with advantages such as biodegradability, biocompatibility, ease of mass production, and reproducibility. The EPS that was isolated from *Rhizobium leguminosarum* bv. *viciae* VF39 is an anionic polysaccharide with a backbone structure consisting of one galactose, five glucose molecules, and two glucuronic acids, along with 3-hydroxybutanoyl, acetyl, and pyruvyl functional groups. Through N-methyl-N′-nitro-N-nitrosoguanidine (NTG) mutagenesis, we isolated and purified a mutant EPS from VF39, VF39 #54, which demonstrated enhanced physicochemical and rheological properties compared to the wild-type VF39. The EPS structure of the VF39 #54 mutant strain showed a loss of glucuronic acid and 3-hydroxybutanoyl groups compared to the wild-type, as confirmed by FT-IR, NMR analysis, and uronic acid assays. The molecular weight of the VF39 #54 EPS was 250% higher than that of the wild-type. It also exhibited improved viscoelasticity and thermal stability. In the DSC and TGA analyses, VF39 #54 had a higher endothermic peak (172 °C) compared to the wild-type (142 °C), and its thermal decomposition point was 260 °C, surpassing the wild-type’s value of 222 °C. Additionally, the VF39 #54 EPS maintained a similar viscosity to the wild-type in various pH, temperature, and metal salt conditions, while also exhibiting a higher overall viscosity. The cytotoxicity test using HEK-293 cells confirmed that the VF39 #54 EPS was non-toxic. Due to its high viscoelastic properties, the VF39 #54 EPS shows potential for use in products such as thickeners, texture enhancers, and stabilizers. Furthermore, its thermal stability and biocompatibility make it a promising candidate for applications in food, pharmaceuticals, and cosmetic formulations. Additionally, its ability to maintain viscosity under varying environmental conditions highlights its suitability for industrial processes that require consistent performance.

## 1. Introduction

Polysaccharides, a class of biopolymers based on monosaccharide structures, have drawn significant attention for their potential applications in films, hydrogels, emulsifiers, and more [1,2,3,4]. Examples of industrially significant polysaccharides include cellulose, alginate, and pullulan [5,6,7]. These materials offer various beneficial properties, such as antioxidant and antibacterial effects, which depend on their monosaccharide composition, bonding patterns, and functional groups [8]. Microbial polysaccharides, a subclass of polysaccharides, are secreted by algae, fungi, and bacteria. Microbial polysaccharides, in particular, offer advantages such as mass production potential and ease of reproduction compared to polysaccharides obtained from animal or plant sources [9,10,11].

Microbial polysaccharides are defined as polysaccharides that can be extracted from microorganisms, and their types include capsular polysaccharides (CPSs), lipopolysaccharides (LPSs), and exopolysaccharides (EPSs) [12]. Microbial EPSs include hyaluronic acid, xanthan, alginate, cellulose, gellan, and succinoglycan [13,14,15,16,17,18]. Recently, numerous research studies of microbial EPSs from various strains have revealed that microbial EPSs have excellent biological and mechanical properties, such as antioxidant, antibacterial, antibiofilm, and rheological properties [19,20,21]. Xanthan is representative of microbial EPSs and has wide applications in industrial fields based on its inherent high thermal stability, water solubility, biocompatibility, and viscosity [22,23,24]. Despite these advantages, microbial EPSs have not been utilized in industrial applications because of their production yields and differences in composition, which change depending on the media conditions. Many researchers have continued to investigate and overcome these shortcomings through strain modification [25], optimization of culture conditions [26,27,28], and metabolic engineering [29].

Nowadays, chemical mutagenesis is often chosen as a method for strain modification, inducing mutations through chemical mutagens such as alkylating agents and azides, including ethyl methanesulfonate (EMS), N-methyl-N′-nitro-N-nitrosoguanidine (NTG), and N-ethyl-N-nitrosourea (ENU) [30]. Chemical mutagenesis can cause random mutations, particularly in transitions and transversions, targeting specific genomic regions with high guanine and cytosine (GC) content or other specific sites in the genome [31]. NTG mutagenesis, which works via alkylating mutations, is considered to be an efficient mutagen due to its high mutation frequency [32]. This method has been applied to various bacterial strains. For example, the NTG treatment of *Nonotus obliquus*, a medicinal fungus, resulted in 32 mutant strains that improved polysaccharide production by about 11-fold [33]. Additionally, EPSs from *Komagataeibacter xylinus* and its mutated strains have exhibited different conformational morphologies and enhanced mechanical properties due to NTG mutagenesis [34].

Among microbial EPSs, those produced by the *Rhizobium* species have been widely studied. The *Rhizobium* species produce EPSs that form biofilms and play a crucial role in the symbiotic relationships with legumes, facilitating nitrogen fixation [35,36]. Notable examples of Rhizobial EPSs include succinoglycan and curdlan, but more recently, there has been a growing interest in the structural characteristics and physicochemical properties of 3-hydroxybutanoylglycan, an EPS produced by *Rhizobium leguminosarum* bv. *viciae* VF39 [37]. The EPS of VF39 is an acidic polysaccharide composed of glucuronic acid, galactose, and glucose, with functional groups such as acetyl, pyruvyl, and 3-hydroxybutanoyl. In previous work, this EPS demonstrated high antioxidant activity and viscoelastic properties, suggesting its significant potential for industrial applications [38].

In this study, we utilized N-methyl-N′-nitro-N-nitrosoguanidine (NTG) mutagenesis to induce mutations in *Rhizobium leguminosarum* bv. *viciae* VF39. We analyzed the EPS produced by the mutant strain and compared it with the wild-type EPS. Our findings revealed structural changes in the mutant EPS, resulting in improved physicochemical properties, such as enhanced viscoelasticity. Through this study, we aim to present a novel approach for developing advanced EPS materials via NTG mutation, with the potential for enhanced industrial applications.

## 2. Materials and Methods

### 2.1. Materials

*Rhizobium leguminosarum* bv. *viciae* VF39 (VF39) was supplied by the Microbial Carbohydrate Resource Bank (MCRB) at Konkuk University, Seoul, Republic of Korea. All chemical reagents were purchased from Sigma-Aldrich Chemicals Co. (St. Louis, MO, USA).

### 2.2. Rhizobium leguminosarum bv. viciae VF39 Strain Culture and Isolation of VF39 EPS

The VF39 strain was incubated for two days at 28 °C in a seed medium containing 0.1% trace elements. The composition of the seed medium was 5 g/L d-mannitol, 1 g/L l-glutamic acid, 1 g/L dibasic potassium phosphate, 0.2 g/L magnesium sulfate, and 0.04 g/L calcium chloride. After incubation, the VF39 strain was transferred to a production medium with the same composition but containing 10 g/L d-mannitol. The production medium was adjusted to a pH of 7.20 and incubated at 28 °C for five days with shaking at 150 rpm. The culture was then centrifuged at 8000 rpm for 15 min at 4 °C to remove cell pellets. To the resulting VF39 EPS supernatant, three volumes of ethanol were added to precipitate the EPS. The precipitated EPS was dried at 60 °C for 24 h, then dissolved in distilled water and dialyzed in a membrane tube (MWCO 12–14 kDa) against distilled water for three days. The dialyzed EPS was lyophilized for later use.

### 2.3. N-Methyl-N′nitro-N-Nitrosoguanidine (NTG) Mutagenesis

**Figure 1:**
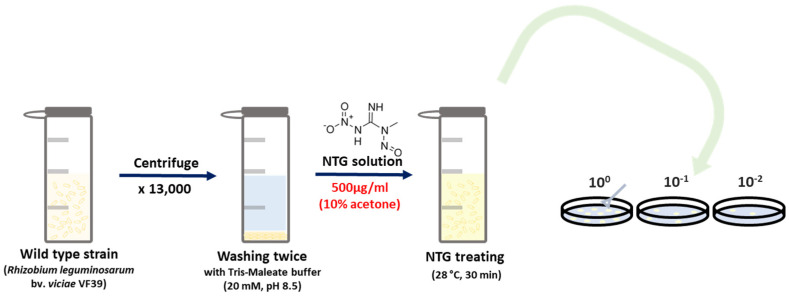
Scheme of VF39 NTG mutagenesis. NTG was dissolved in distilled water with 10% acetone for activation.

In Figure 1, an illustration of the general process of NTG mutation is presented. Briefly, the VF39 strain was cultured for two days and then centrifuged at 1000 rpm for 3 min. After centrifugation, the cell pellet was washed twice with a 20 mM solution of Tris-Maleate buffer (pH 8.5). The NTG solution, prepared at a concentration of 0.5 mg/mL in 10% acetone, was activated by shaking at 28 °C and 200 rpm for 30 min. Mutagenesis was terminated by treating the cells with sodium thiosulfate in a saline solution [39]. The cell suspension was then diluted to a concentration of 1/10^−6^, 1/10^−7^, 1/10^−8^ and spread on a GMS medium containing 1.5% agar. The plates were incubated at 28 °C for two days. Colonies that appeared larger and had more moisture were selected, and the mutant strain was designated VF39 #54.

### 2.4. Nuclear Magnetic Resonance (NMR) Spectroscopy

The ^1^H NMR spectrum was acquired at 600 MHz using a Bruker Avance spectrometer (Bruker, Karlsruhe, Germany). For the ^1^H NMR, 10 mg of VF39 EPS was dissolved in 1 mL of D_2_O. The VF39 EPS was measured at 25 °C. The VF39 #54 EPS was measured at 60 °C. Additionally, the ^13^C NMR spectrum and heteronuclear single quantum coherence (HSQC) spectrum were obtained at 600 MHz using a Bruker Avance III-600 spectrometer (Bruker, Karlsruhe, Germany). For the ^13^C NMR, 80 mg of the VF39 EPS was dissolved in 1 mL of D_2_O at 60 °C and measured at 25 °C.

### 2.5. Fourier Transform Infrared (FT-IR) Spectroscopy

The FT-IR spectrum of the VF39 EPS was recorded using an FT-IR spectrometer in ATR mode (resolution 0.5 cm^−1^, Bruker, Bremen, Germany) over a wavenumber range of 4000–650 cm^−1^. Each spectrum was obtained from lyophilized samples with 16 scans.

### 2.6. Glucuronic Acid Concentration Assay

The uronic acid concentrations were determined using the m-hydroxydiphenyl method, as described by Blumenkrantz and Asboe-Hansen [40]. Briefly, 0.2 mL of VF39 EPS solutions (ranging from 0.0625 to 0.5 mg/mL) were mixed with 1.2 mL of sulfuric acid/tetraborate, and the mixtures were heated in a water bath at 100 °C for 5 min. After cooling in an ice bath, 20 μL of m-hydroxydiphenyl reagent was added. The tubes were shaken, and the absorbances measured at 520 nm within 5 min.

### 2.7. Differential Scanning Calorimetry (DSC)

A DSC analysis was performed using a Discovery DSC 2500 (TA Instruments, New Castle, DE, USA). Dried samples (10 mg) were placed in a sealed aluminum pan and heated under a nitrogen atmosphere. The heat flow was recorded at a scan rate of 10 °C/min over a temperature range of 20–200 °C.

### 2.8. Thermal Gravimetric Analysis (TGA)

A TGA was conducted using an STA 449 F3 Jupiter (NETZSCH, Weimar, Germany). Dried samples (10 mg) were prepared, and the weight loss was monitored under a nitrogen atmosphere as the temperature increased from 20 °C to 600 °C at a rate of 10 °C/min.

### 2.9. Molecular Weight by Gel Permeation Chromatography (GPC)

GPC was performed using a Waters 2414 refractive index detector (Waters Corporation, Milford, MA, USA) and a Waters 1525 binary pump (Waters Corporation, USA) with NaNO_3_ solution in distilled water as the mobile phase. The column comprised Waters Ultrahydrogel Linear (Waters Corporation, USA), 500, 250, and 120. Sodium nitrate (0.02 N) was used as the elution solvent, and pullulan served as the calibration standard (pullulans 642,000, 334,000, 201,000, 110,000, 49,400, 2200, 9400, 6300 peak molecular weight). The flow rate was set at 0.8 mL/min. GPC was performed in the instrumental analysis center of Kyungpook National University.

### 2.10. Rheological Measurements

The viscoelastic properties were evaluated using a DHR-2 rheometer (TA Instruments, USA). The sample solutions were prepared at varying concentrations (2 wt%, 4 wt%, 6 wt%, 8 wt%, 10 wt%) of the VF39 EPS. Except for viscosity measurements according to concentration, the 2 wt% solution was commonly used for viscosity measurements according to temperature, pH, and salt conditions. Viscosity tests were conducted at shear rates ranging from 0.01 to 1000 s^−1^. Temperature studies were performed at 25 °C, 35 °C, 45 °C, 55 °C, 65 °C, and 75 °C [39]. pH studies were conducted at pH levels of 2, 5, 7, 9, and 12, while viscosity measurements in salt conditions were tested using 0.25 M of NaCl, KCl, MgCl_2_, and CaCl_2_ [41]. For the temperature sweep tests, the temperature ranged from 25 °C to 75 °C with a heating rate of 10 °C/min, and the sample pH was adjusted to 7.0 [39]. The amplitude sweep test was measured over a range of 0.1–1000%, with the frequency kept constant. The frequency sweep test was conducted over a range of 0.1–600 Hz at a constant strain of 0.5%.

### 2.11. Gelation Test by Metal Cations

Various metal ion solutions (NaCl, KCl, CaCl_2_, MgCl_2_, ZnCl_2_, FeCl_2_, FeCl_3_, CuCl, CuCl_2_, AlCl_3_, CrCl_3_) were prepared at a concentration of 0.25 M in distilled water. The VF39 EPS solutions (1% *w*/*v*, aqueous) were vortexed with an equal volume of each metal ion solution for 2 min. All samples were then left undisturbed for 24 h at 25 °C [38].

### 2.12. Antioxidant Test

The antioxidant activity of each sample was evaluated based on their scavenging efficiency against the 2,2-diphenyl-1-picrylhydrazyl (DPPH) free radical [42]. A mixture of 2.0 mL deionized water, 1.0 mL VF39 EPS sample (at concentrations of 1, 2, 3, and 4 mg/mL), and 0.4 mL DPPH solution (0.5 mM) was prepared. The mixtures were incubated in the dark at 30 °C for 30 min, after which the absorbances were measured at 517 nm. The DPPH radical scavenging activity was determined as follows:

$$\mathrm{DPPH}\;\mathrm{radical}\;\mathrm{scavenging}\;\mathrm{activity}\;(\%)=\frac{A_{0}-A_{s}}{A_{0}}\times 100\%$$

where $A_{0}$ represents the absorbance of the control, and $A_{s}$ is the absorbance of the samples. Each test was performed in triplicate.

### 2.13. Cell Cytotoxicity

The cytotoxicity of the VF39 EPS was evaluated using WST-8 assays. Human embryonic kidney 293 cells (HEK-293, Korean Cell Line Bank, Seoul, Republic of Korea) were seeded into 96-well culture plates containing a Minimum Essential Medium (MEM, WELGENE, Gyeongsan-si, Republic of Korea) supplemented with 10% fetal bovine serum and 1% penicillin/streptomycin. The VF39 EPS was added to the wells at concentrations of 0.25, 0.5, and 1 mg/mL, and the plates were incubated at 37 °C in an atmosphere containing 5% CO_2_. The positive control comprised 10% (*v*/*v*) dimethyl sulfoxide (DMSO) dissolved in MEM. After 48 h of incubation, the MTT assay reagent (QuantiMax, BIOMAX, Seoul, Republic of Korea) was added to each well, and absorbance was measured at 550 nm [43]. Cell viability was determined using the following formula:

$$\mathrm{Cell}\;\mathrm{viability}\;(\%)=\frac{A_{s}-A_{0}}{A_{c}-A_{0}}\times 100\%$$

$A_{s}$ represents the absorbance value of the cells cultured with the EPS sample, while $A_{c}$ is the absorbance value of the control cells. $A_{0}$ is the blank solution containing only the minimal essential medium, MTT assay solution, and solubilization solution. All assays were performed in triplicate for each sample.

## 3. Results and Discussion

### 3.1. Isolation of VF39 Wild-Type and #54 EPSs

**Figure 2:**
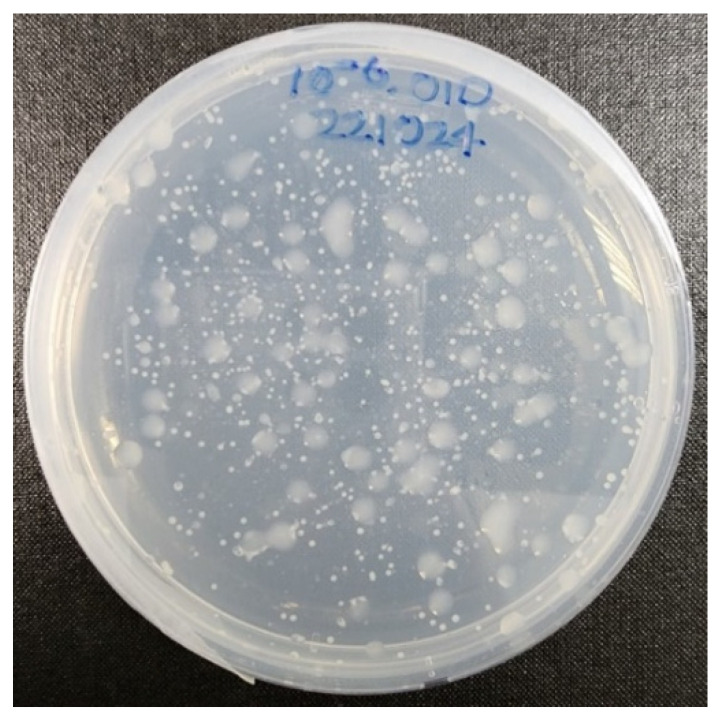
Cultured mutant strain colonies from the VF39 wild-type after NTG mutagenesis.

The isolation and extraction of the EPSs were carried out using both the VF39 wild-type strain and the NTG mutant #54 strain. After treating the VF39 strain with NTG, randomly mutated colonies were cultured, as shown in Figure 2. Some mutant colonies formed larger and wider biofilms compared with the wild-type under the same conditions. Large, sticky, and moist colonies were selected for further analysis. These selected colonies were individually cultured to produce EPSs from the mutant strains. Both the selected mutant strains and the VF39 wild-type strain were then cultured again to produce the EPSs. The cultured medium was centrifuged to separate the cells, and the EPS present in the supernatant was purified using ethanol precipitation and dialysis. The purified EPS solutions were freeze-dried, yielding EPSs from the VF39 wild-type and the mutant VF39 #54 strains, which were used for further analysis.

### 3.2. Characterization of VF39 Wild-Type and #54 EPSs

**Figure 3:**
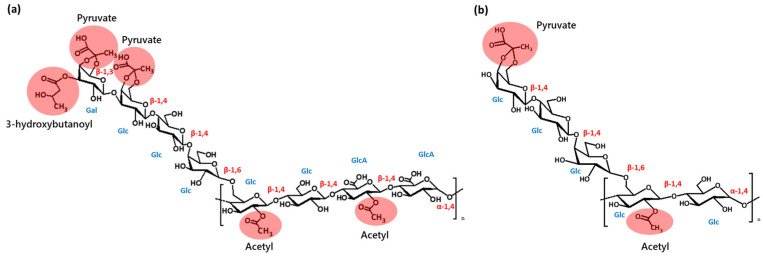
Structure of the VF39 EPS (**a**) and #54 EPS (**b**). The attached functional groups, 3-hydroxybutanoyl, pyruvate, and acetyl, are highlighted with red circles.

In Figure 3a, the structure of the VF39 wild-type EPS, as previously reported [37,38], was determined through FT-IR and NMR analyses. The EPS structure consists of two D-glucuronic acids, five D-glucose molecules, one D-galactose, one acetyl, two pyruvyl, and one 3-hydroxybutanoyl functional group. This acidic polysaccharide is composed of repeating octasaccharide units with β-1,3, β-1,4, β-1,6, and α-1,4 glycosidic bonds. A similar structure was previously reported in the EPS of *Rhizobium leguminosarum* strains [44]. The structure and properties of the NTG mutant VF39 EPS were compared with those of the VF39 wild-type strain using GPC, FT-IR, NMR, and glucuronic acid assays.

#### 3.2.1. Fourier Transform Infrared (FT-IR)

**Figure 4:**
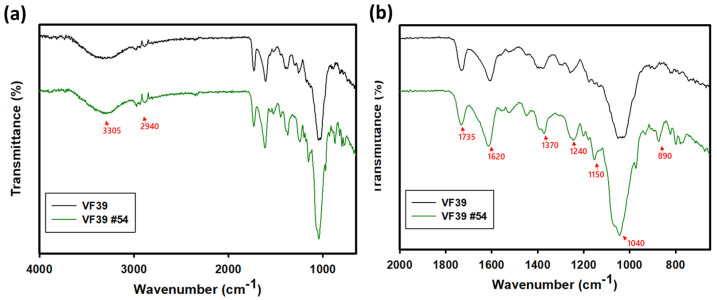
FT-IR spectra of the VF39 EPS and VF39 #54 EPS (**a**) and expanded spectra of wavelength 2000~600 cm^−1^ (**b**).

To characterize the EPSs of the VF39 wild-type and #54, FT-IR spectra were analyzed. As shown in Figure 4 and Table 1, the two EPS samples exhibited similar FT-IR spectra, showing peaks in common with the previously studied *Rhizobium leguminosarum* strain [45]. The VF39 EPS exhibited both α- and β-glycosidic linkages, with corresponding peaks at 1150 cm^−1^ and 860 cm^−1^. Notably, the VF39 #54 EPS showed a stronger intensity at 1150 cm^−1^, suggesting a higher presence of α-glycosidic linkages [38].

#### 3.2.2. Nuclear Magnetic Resonance (NMR) Spectroscopic Analysis

**Figure 5:**
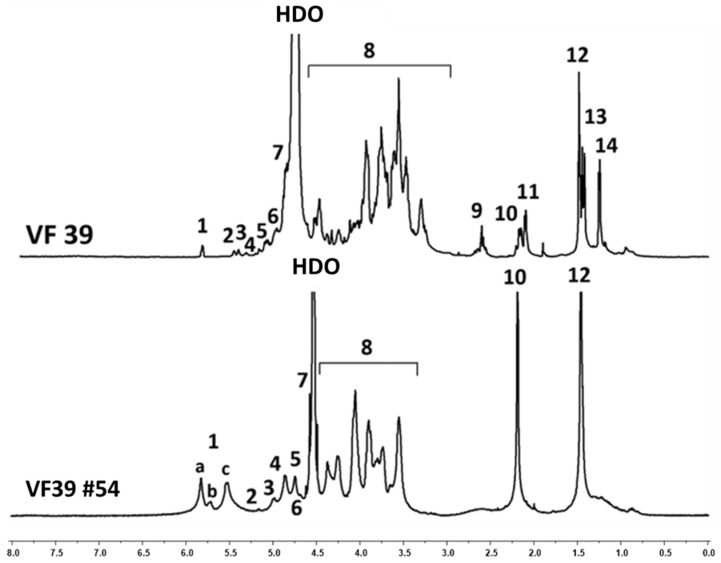
The ^1^H NMR of the VF39 and #54 EPSs. The ^1^H NMR spectra was measured using a 10 mg/mL concentration of sample solutions dissolved with D_2_O (99%) at 60 °C.

In the ^1^H NMR spectra at Figure 5, both samples represented the structure of the carbohydrate backbone. The ring protons that form the polysaccharide could be observed between 3.3 and 4.5 ppm [46]. Peaks corresponding to the linkages between the monosaccharide units of the polysaccharide were as follows: protons related to the α-glycosidic bond appeared after 5.0 ppm, while protons associated with the β-glycosidic bond appeared in the 4.5–5.0 ppm range [46]. Additionally, the methyl peak of the pyruvyl group, commonly found in rhizobial polysaccharides, appeared between 1.4 and 1.5 ppm, and the methyl peak of the acetyl group was observed between 2.1 and 2.2 ppm [47].

However, some structural differences between the EPSs of the wild-type and the NTG mutant strain were confirmed through ^1^H NMR analysis [46]. In the VF39 wild-type, the presence of acetyl groups on d-glucose and d-glucuronic acid was identified by the split signal peaks related to the o-acetyl residue at δ 2.11 and 2.18 ppm. A similar split signal pattern was observed for the pyruvyl functional group. In the wild-type, the pyruvyl residues were linked to both d-glucose and d-galactose, with the latter containing 3-hydroxybutanoyl. Consequently, the pyruvyl residue appeared as two distinct peaks: δ 1.47 ppm (linked to d-galactose) and δ 1.44 ppm (linked to d-glucose). The 3-hydroxybutanoyl group was identified through the -CH_2_- peak of methylene at δ 2.63 ppm and the -CH_3_ methyl peak at δ 1.27 ppm. In contrast, the NMR spectra of the VF39 mutant strain #54 exhibited distinct differences in the functional group peaks compared to the wild-type structure. Most notably, the absence of the 3-hydroxybutanoyl functional group was confirmed by the disappearance of its characteristic peaks. Additionally, the signals for both the acetyl and pyruvyl residues were simplified into singlet peaks: the acetyl residue appeared at δ 2.14 ppm, and the pyruvyl residue at δ 1.47 ppm [48]. The absence of the acetyl peak at δ 2.18 ppm indicated that the acetyl group on d-glucuronic acid was no longer present in the mutant. Furthermore, the pyruvyl signal at δ 1.44 ppm, corresponding to pyruvyl attached to galactose (which was linked to 3-hydroxybutanoyl in the wild-type), disappeared. This signal was integrated into the peak at δ 1.47 ppm, suggesting that the chemical environment had changed due to the loss of the 3-hydroxybutanoyl functional group. Based on the NMR data, the EPS structure of the mutant strain #54 is depicted in Figure 3b. he assignments of the NMR peaks are designated in Table 2.

#### 3.2.3. Gel Permeation Chromatography (GPC)

The molecular weight of each EPS sample was determined using GPC analysis, as shown in Table 3. This significant increase in molecular weight is likely responsible for the greater stickiness and higher viscosity observed in the mutant strain colonies. In a similar case, there was a report that the molecular weight of the EPS of *Agrobacterium* 19,358 strain increased through NTG mutation [39].

#### 3.2.4. ^13^C NMR of the VF39 Wild-Type and VF39 #54 EPSs

**Figure 6:**
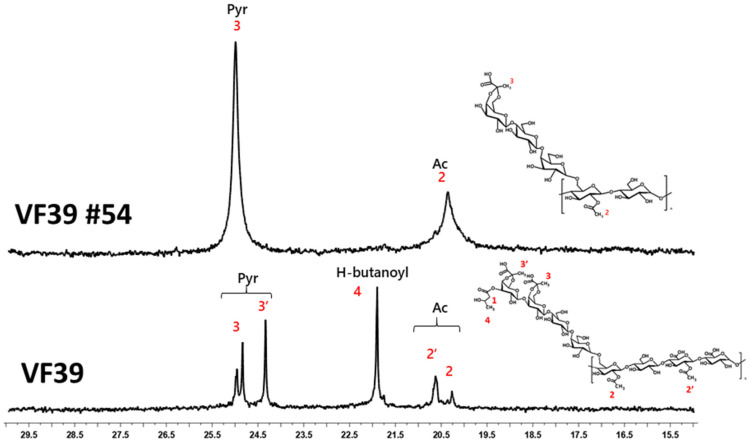
^13^C NMR methyl peak of the VF39 and VF39 #54 EPSs. The methyl peaks of acetyl (2,2′), pyruvate (3,3′), and 3-hydroxybutanoyl (4) are shown in the VF39 EPS spectrum. The VF39 #54 EPS shows only the acetyl (2) and pyruvyl (3) peaks.

In Figure S1, the ^13^C NMR spectrum for the VF39 #54 showed the absence of the methylene peak at 45 ppm, which corresponds to the 3-hydroxybutanoyl functional group in the wild-type EPS [49]. Additionally, as shown in Figure 6, the methyl peak of 3-hydroxybutanoyl was also missing in the VF39 #54, confirming that this functional group had been lost through mutation. Furthermore, the methyl peaks of the pyruvyl and acetyl functional groups in the VF39 #54 appeared as singlets at 20 ppm and 25 ppm, respectively, in contrast to the wild-type [49,50]. This indicated a reduction in the pyruvyl and acetyl functional groups compared to the wild-type, as illustrated in the structure shown in Figure 3b.

#### 3.2.5. HSQC NMR of the VF39 Wild-Type and #54 EPSs

**Figure 7:**
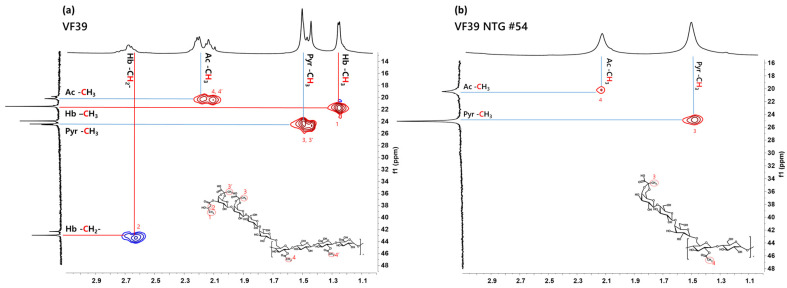
HSQC functional group spectra of the VF39 and VF39 #54 EPSs with D_2_O (99%). Peaks of NMR data revealed 3-hydroxylbutanoyl (1,2), pyruvyl (3,3′), and acetyl (4,4′) groups of EPS samples.

HSQC NMR was used to identify the peaks corresponding to functional groups by correlating the ^1^H NMR and ^13^C NMR spectra. As shown in Figure S2, the carbohydrate backbone structure of the VF39 #54 EPS exhibited fewer signals compared to the VF39 wild-type. This reduction was likely due to a decrease in the number of functional groups and monomers that constitute the backbone of the VF39 #54 EPS. Additionally, Figure 7 provides a zoomed-in view of the functional group region in Figure S2. In this result, no signal for the 3-hydroxybutanoyl functional group was detected in the VF39 #54 EPS [38]. Furthermore, changes were observed in the pyruvyl and acetyl peaks of the VF39 #54 EPS. While two distinct peaks were present for the pyruvyl and acetyl groups in the VF39 wild-type, only one peak was observed for each group in the VF39 #54 EPS. This indicated a reduction in the number of pyruvate and acetyl groups in the VF39 #54 EPS.

#### 3.2.6. Glucuronic Acid Concentration Analysis

**Figure 8:**
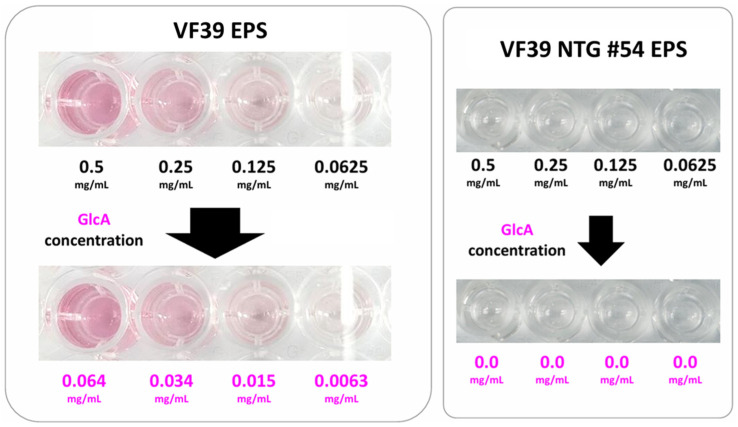
Glucuronic acid concentration assay with the *m*-hydroxydiphenyl method. No glucuronic acid was detected in the VF39 #54 EPS.

In Figure 8, to evaluate the presence or absence of glucuronic acid in the EPS structure of the VF39 mutant #54 strain, suggested by NMR analysis, a uronic acid concentration analysis was conducted. The concentration of glucuronic acid was determined by measuring absorbance at 520 nm using the m-hydroxydiphenyl method [40]. In the VF39 wild-type EPS, an absorbance at 520 nm was observed, indicating the presence of glucuronic acid, with concentrations corresponding to the EPS solution concentration. However, glucuronic acid was not detected at any EPS concentrations in the VF39 #54. This confirmed the absence of glucuronic acid in the mutant EPS, consistent with the changes in the acetyl residue peaks observed in the NMR spectra.

#### 3.2.7. Thermal Gravimetric Analysis (TGA) and Differential Scanning Calorimetry (DSC)

**Figure 9:**
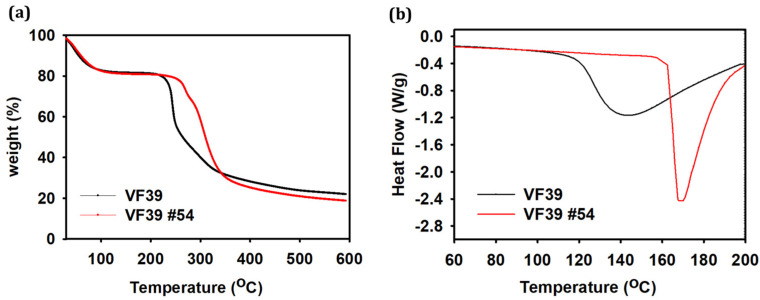
(**a**) TGA and (**b**) DSC curves of the VF39 and VF39 #54 EPSs.

The thermal properties of polysaccharides were analyzed in Figure 9, to assess their thermal stability, which is closely linked to their potential for industrial applications [51,52]. A thermogravimetric analysis (TGA) was used to examine degradation as a function of temperature, and differential scanning calorimetry (DSC) was used to analyze the heat flow within the sample and determine the endothermic point.

The TGA results showed that the mass loss from the VF39 EPS between 20 °C and 100 °C was due to water evaporation. At 600 °C, the VF39 EPS showed 79% weight loss, and 21% of the residual weight was measured. The VF39 #54 EPS showed 81% weight loss, and 19% of the residual weight was measured. Additionally, the temperature at which rapid thermal decomposition began for the VF39 wild-type EPS was 222 °C. In contrast, the VF39 #54 EPS exhibited thermal decomposition starting at a higher temperature of 260 °C, indicating greater thermal stability for the NTG mutant strain.

DSC measurements were conducted for a further comparison of thermal stability. The endothermic point, where rapid changes in heat flow occur due to molecular structure changes, was identified [53,54]. The wild-type VF39 EPS had an endothermic point at 142 °C, while the VF39 #54 EPS showed an endothermic point at 172 °C, suggesting that structural disordering due to heat transfer occurred at a higher temperature. These DSC results, which aligned with the TGA findings, confirmed that the EPS of the NTG mutant strain has a greater thermal stability than the wild-type.

### 3.3. VF39 Wild-Type and #54 EPS Rheological Measurements

The rheological properties of polysaccharides offer significant potential for commercialization, serving as key indicators for their use as industrial materials in applications such as thickeners, stabilizers, texture enhancers, and gelling agents [9,55]. These properties are essential for the development of biomaterials that can maintain high viscoelasticity, even under challenging conditions such as high temperatures and varying pH levels. To explore their potential, the rheological properties of the VF39 EPS samples were measured and analyzed under different conditions, including concentration, temperature, and pH. The VF39 EPS exhibited pseudoplastic viscoelasticity, which is attributed to intermolecular interactions such as hydrogen bonding between carboxyl and alcohol groups. These viscoelastic properties were compared to those of xanthan gum, a commonly used polysaccharide [56].

#### 3.3.1. Viscosity Measurements of the VF39 Wild-Type and #54 EPSs

**Figure 10:**
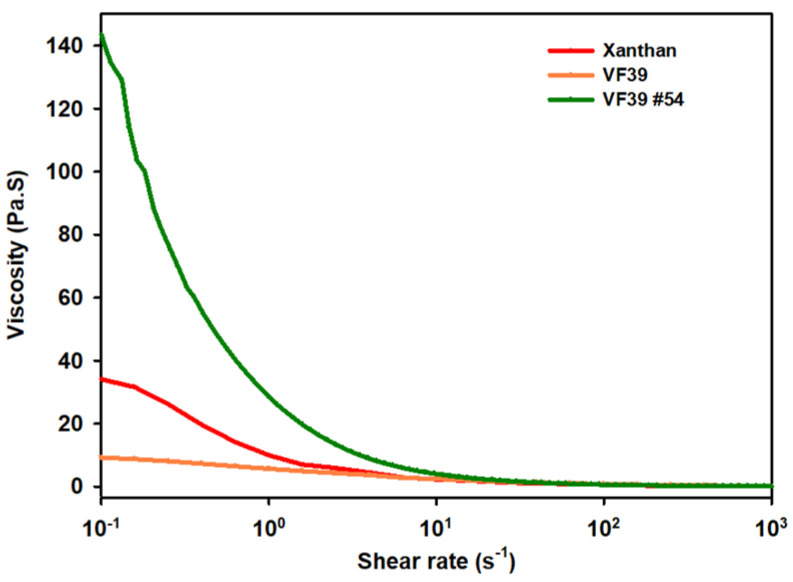
Viscosity of the VF39 wild-type and #54 EPSs with a 2% concentration of solution. Measurements were performed at 25 °C.

In the viscosity measurement, the viscoelasticity of the VF39 EPS sample was compared to that of xanthan gum. The results, measured at a concentration of 2% and a shear rate ranging from 0.1 to 1000 s^−1^, are shown in Figure 10. The VF39 #54 EPS exhibited higher viscoelasticity than both the wild-type VF39 EPS and xanthan gum, indicating greater viscosity. Thus, the VF39 #54 EPS, enhanced through mutation, demonstrated a higher potential for industrial applications where viscoelastic properties are crucial.

#### 3.3.2. Viscosity Measurements with Concentration, Temperature, pH, and Metal Salts

To industrially utilize viscoelastic materials, it is essential to maintain their properties under various environmental conditions. Therefore, the VF39 wild-type and #54 EPS samples were tested under different concentrations, temperatures, pH levels, and metal salt conditions.

**Figure 11:**
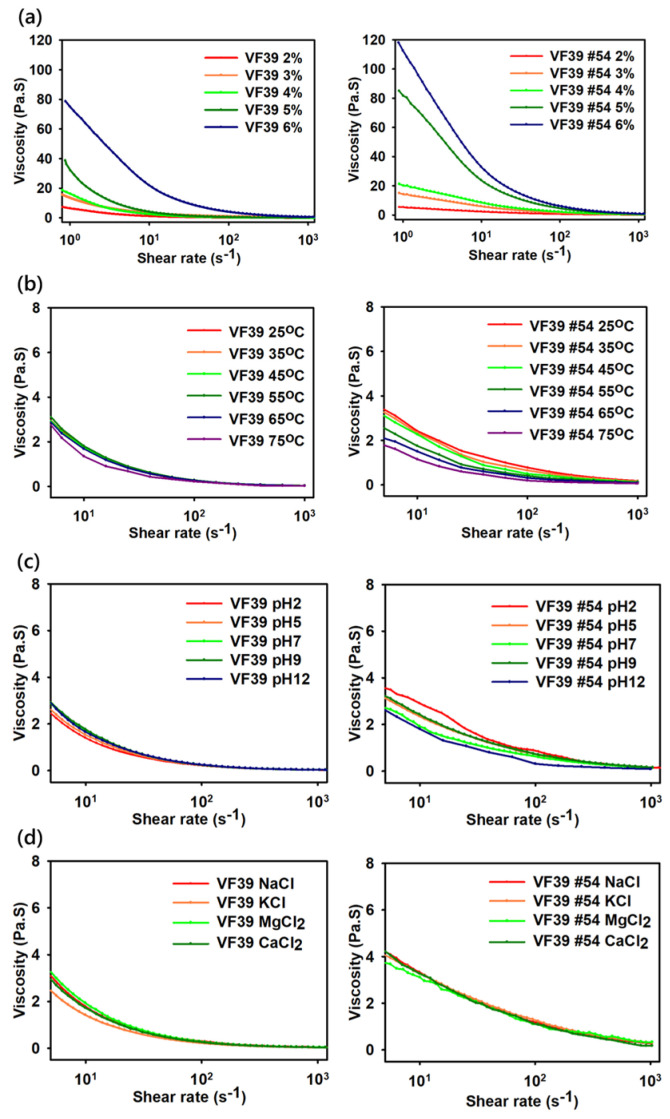
Viscosity of the VF39 wild-type and #54 with different concentrations (**a**), temperature (**b**), pH (**c**), and metal salts (**d**).

**Figure 12:**
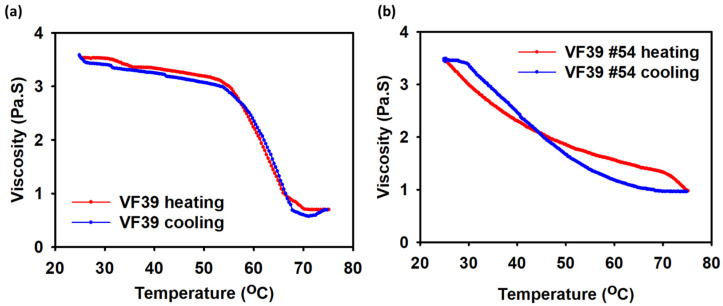
Rheological measurements of (**a**) the VF39 wild-type EPS and (**b**) the VF39 #54 EPS with heating and cooling from 25 °C to 75 °C at 2 wt% concentration with shear rate of 10 s^−1^.

First, as shown in Figure 11a, both samples exhibited increased viscosity with increasing concentration. As the EPS concentration increased from 2 wt% to 6 wt%, the viscosity followed a similar trend to other exopolysaccharides such as xanthan and succinoglycan [39,57]. Among the two samples, the VF39 #54 EPS demonstrated a higher viscosity than the wild-type EPS at the same concentration. In Figure 11b, viscosity was measured as the temperature increased from 25 °C to 75 °C. As expected, the viscosity of both EPS samples decreased with rising temperature [58]. However, this decrease did not indicate a decomposition of the EPS. Figure 12 shows that the viscosity of the VF39 EPS samples recovered after the heating and cooling processes, suggesting that while intermolecular interactions weaken at high temperatures, the samples did not break apart. Notably, the viscosity of the VF39 #54 EPS remained above 1 Pa·s at a shear rate of 10 s^−1^ even at 75 °C, indicating that the VF39 #54 EPS maintained a higher viscosity under elevated temperature conditions compared to the wild-type EPS. Additionally, the preservation of the EPS structure after the heating and cooling process was confirmed using FT-IR. An EPS sample 2 wt% solution was prepared by heating to 80 °C and freeze-drying the cooled sample. In Figures S3 and S4, the FT-IR spectra appeared similar even after the heating process, showing that the structure of the sample was not destroyed. In Figure 11c,d, viscosity was measured under varying pH and metal salt conditions. The viscosity of the EPS samples remained relatively stable across the pH range of 2.0 to 12.0, indicating greater stability to pH changes than xanthan gum. For the metal salt conditions, four ion solutions (NaCl, KCl, MgCl_2_, and CaCl_2_) at 0.25 M were used. All VF39 EPS samples showed stability under these salt conditions, with no significant changes in viscosity observed. In salt conditions, the VF39 EPS samples showed viscosity changes of less than 5%, which indicated a higher retention capacity compared to xanthan, whose viscosity decreased in salt conditions [59,60].

#### 3.3.3. Frequency Sweep Test and Amplitude Sweep Test

**Figure 13:**
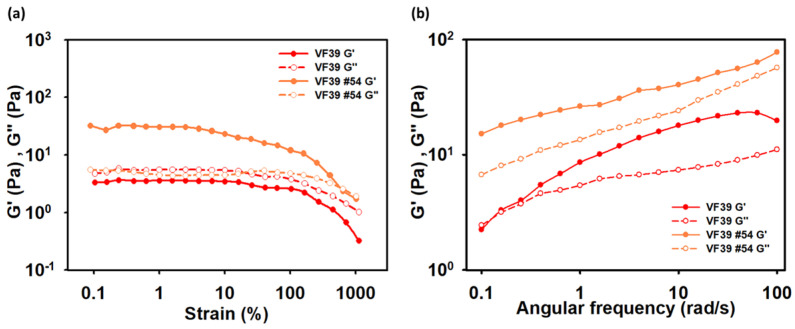
Rheological measurements with (**a**) frequency sweep test and (**b**) amplitude sweep test with a 2 wt% concentration at 25 °C.

Rheological measurements were conducted using a 2 wt% concentration of the EPS sample solution. The physical properties were analyzed by measuring the strain in proportion to the applied stress and observing changes in material behavior accordingly. In Figure 13a, the linear viscoelastic region (LVE) of the VF39 EPS samples was determined through amplitude strain testing. The storage modulus (G′) and loss modulus (G″) of both the VF39 wild-type and #54 EPSs were measured. The results indicated that, unlike the wild-type, the VF39 #54 EPS exhibited a stronger G′ than G″, suggesting weak gel-like properties [61]. In Figure 13b, the frequency sweep test showed that the VF39 EPS exhibited elastic properties at low frequencies, with G′ being greater than G″. However, at higher frequencies, the increase in G″ indicated more viscous behavior. In contrast, the VF39 #54 EPS exhibited elastic properties across all frequency ranges, indicating a higher modulus value and stronger physical properties compared to the wild-type EPS.

### 3.4. Metal Chelating Gelation by Metal Cations

**Figure 14:**
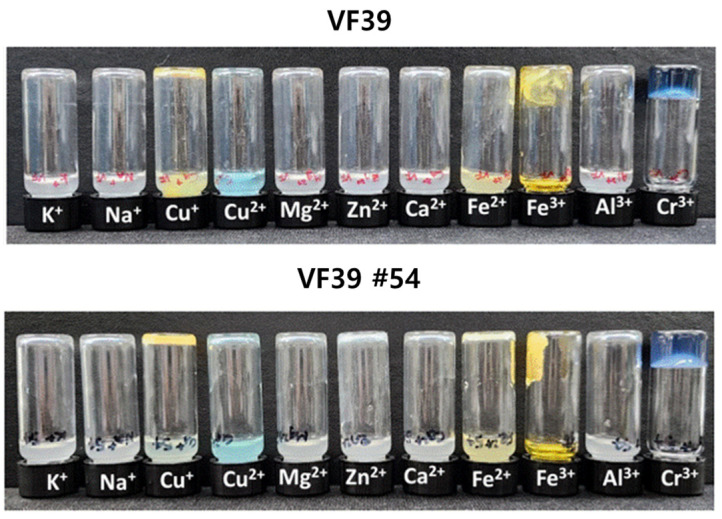
Gelation of the VF39 and VF39 #54 EPSs with metal chelation.

Some polysaccharides form hydrogels with specific metals through ion-based interactions such as chelation [62,63]. For a hydrogel to form between a metal cation and an anionic polysaccharide, the polysaccharide must have a three-dimensional structure that effectively coordinates with the metal cation. A chelation test was conducted to evaluate the potential for chelation and hydrogel formation of the VF39 EPS with metals, using a 0.25 M metal ion solution. As shown in Figure 14, both the VF39 wild-type and #54 EPSs exhibited the same behavior. Both types of EPS formed hydrogel structures with two metals, Fe^3+^ and Cr^3+^. The metal chelation test results indicated that the VF39 EPS formed hydrogel structures due to the interaction of its carboxyl groups and the unique structure of the EPS with the introduced metal ions [64].

### 3.5. DPPH Radical Scavenging Antioxidant

**Figure 15:**
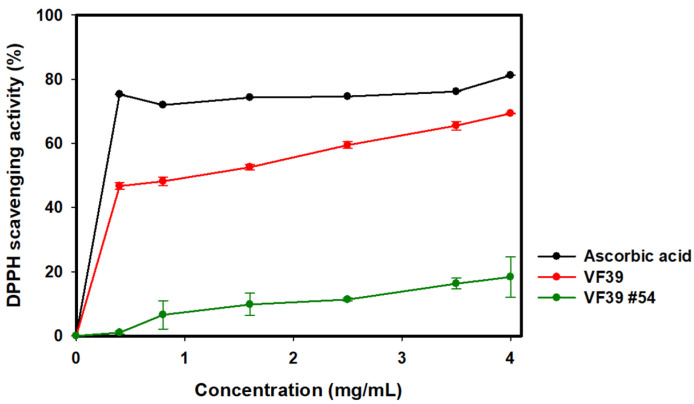
Antioxidant activity of the VF39 EPS samples with DPPH radical scavenging activity test.

The antioxidant effect was evaluated using the DPPH radical scavenging activity test. The degree of radical scavenging was determined by measuring absorbance at 517 nm after reacting the sample solution with DPPH. It has been reported that the radical scavenging activity of the EPS is influenced by the presence of carboxyl groups with anionic charges [65]. As shown in Figure 15, the VF39 wild-type EPS exhibited a scavenging activity of 63% at 4 mg/mL, which is attributed to the functional groups containing glucuronic acid and carboxyl groups. The wild-type EPS demonstrated an antioxidant effect comparable to ascorbic acid. However, the mutant EPS showed a significantly reduced antioxidant effect, likely due to the absence of glucuronic acid and other functional groups containing anionic carboxyl groups in its structure. These results confirm that the EPS structure of the mutant strain differs from that of the wild-type, as reflected in its diminished antioxidant activity.

### 3.6. Cell Cytotoxicity of VF39 Wild-Type and Mutant EPS

**Figure 16:**
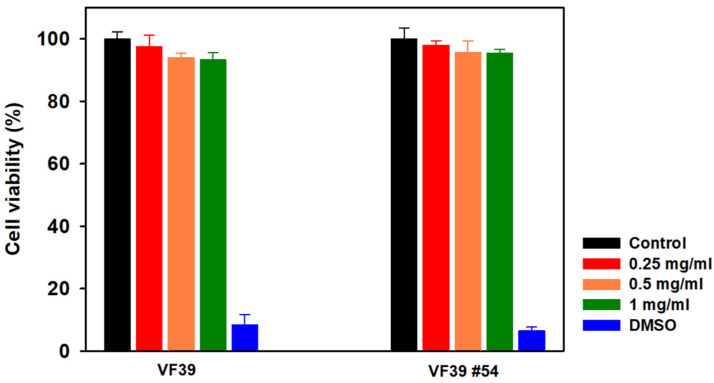
Cell cytotoxicity test of the VF39 EPS samples with HEK293 cells. DMSO was used as a negative control.

A cell cytotoxicity test was conducted to assess the cytotoxicity of the VF39 EPS and the mutant strain using an MTT assay kit with HEK293 cells. The VF39 EPS was applied to HEK293 cells at concentrations of 0.25, 0.5, and 1.0 mg/mL, and absorbance was measured at 550 nm after 48 h. DMSO was used as a negative control. As shown in Figure 16, none of the VF39 EPS samples exhibited cytotoxicity. The VF39 wild-type EPS showed cell viability rates of 97.44%, 93.97%, and 93.22% at concentrations of 0.25, 0.5, and 1.0 mg/mL, respectively. Similarly, the VF39 #54 EPS exhibited cell viability rates of 97.93%, 95.49%, and 95.38% at the same concentrations. Therefore, both the VF39 wild-type EPS and VF39 #54 EPS were confirmed to be non-cytotoxic.

## 4. Conclusions

We isolated the VF39 #54 strain, which exhibited improved viscoelasticity, through the NTG-induced mutation of VF39. The isolated VF39 #54 EPS was structurally characterized using FTIR, GPC, NMR, and uronic acid assays. Additionally, its physicochemical properties were analyzed through DSC, TGA, rheology, metal chelating gelation tests, and cell cytotoxicity assays. Structural analysis and metal gelation tests revealed that the VF39 #54 EPS exhibited changes in functional groups compared to the wild-type EPS. Furthermore, the DSC and TGA results demonstrated higher thermal stability and enhanced viscoelastic properties. The VF39 #54 EPS displayed superior viscoelastic properties compared to the wild-type and was able to maintain these properties even under various environmental conditions. The VF39 #54 EPS showed a lower antioxidant activity than the wild-type EPS, which indicated that the glucuronic acid unit and pyruvyl functional group of the wild-type EPS were lost, and the carboxyl group was reduced. In addition, the VF39 #54 EPS showed no cytotoxicity in the cell cytotoxicity test, just like the wild-type EPS.

These findings indicate that the VF39 #54 EPS has significant industrial potential in applications such as food and cosmetics, where viscoelasticity and thermal stability are critical. Its enhanced properties make it suitable for use as a thickener, stabilizer, or gel agent in formulations requiring consistent performance under diverse conditions. Additionally, this study highlights a promising approach to developing biomaterials with improved properties through targeted strain mutations.

## Figures and Tables

**Table 1:** FT-IR peak analysis of the VF39 wild-type and #54 EPSs.

Wave Number (cm^−1^)	Structural Designation
3305	O-H stretching vibration
2940	-CH_2_, -CH_3_ asymmetric stretching vibration
1750	C=O stretching vibration of carboxyl group
1620	C=O asymmetric stretching vibration of carboxyl group
1370	Symmetric stretching vibration of -COO- group
1240	C-O-C stretching vibration of glycosidic linkage
1150	Stretching vibration of α-glycosidic bond
1040	C-O-C asymmetric stretching vibration of glycosidic linkage
890	Stretching vibration of β-glycosidic bond

**Table 2:** Peak analysis from ^1^H NMR of the VF39 wild-type and #54 EPSs.

Signal Number	ppm	Proton	Assignment
1a	5.99	H-1	α-4,6-Glc-2Ac
1b	5.89	H-1	α-4,6-Glc-3Ac
1c	5.75	H-1	α-4,6-Glc
2	5.38	H-2	α-4,6-Glc-2Ac
3	4.92	H-2	β-4-Glc-2Ac
4	4.80	H-1	β-4,6-Glc-3
5	4.65	H-1	β-pyranose
6	4.59	H-1	Unidentified
7	4.48	H-1	β-anomeric pyranoside
8		Ring protons	Ring protons of the β-linked residues (except anomeric protons of H-1)
9	2.63	CH_2_	3-Hydroxybutanoyl-6 on β4,6-Gal-3
10	2.18	OAc	Glc-3Ac
11	2.11	OAc	GlcA-3Ac
12	1.47	CH_3_ of pyruvic acetal	Glc-5,6 pyr
13	1.44	CH_3_ of pyruvic acetal	Gal-5,6 pyr
14	1.27	CH_3_	Gal-3-Hydroxybutanoyl-6

**Table 3:** Substituent ratio analysis from ^1^H NMR peak intensity and molecular weight of the VF39 wild-type and #54 EPSs.

Sample	Molar Ratio of VF39’s Substituent			Number Average Molecular Weight (Mn)	Weight Average Molecular Weight (Mw)	Peak Molecular Weight (Mp)	Polydispersity
	3-Hydroxyl Butanoyl	Acetate	Pyruvate				
VF39	0.32	1.34	2	161,880	327,400	ND	2.022
VF39 #54	-	0.72	1	301,170	363,100	515,140	1.205

## Data Availability

The original contributions presented in the study are included in the article/Supplementary Materials, further inquiries can be directed to the corresponding author.

## Supplementary Materials

The following supporting information can be downloaded at: https://www.mdpi.com/article/10.3390/polym16223179/s1, Figure S1: ^13^C NMR of VF39, and #54 EPS. ^13^C NMR spectra was measured at 80 mg/mL concentration of sample solutions dissolved with D_2_O (99%); Figure S2: HSQC NMR spectra of VF39, and VF39 #54 EPS. HSQC NMR spectra was measured at 80 mg/mL concentration of sample solutions dissolved with D_2_O (99%); Figure S3: FT-IR spectra of VF39 EPS, and VF39 EPS after heating and cooling sample; Figure S4: FT-IR spectra of VF39 #54 EPS, and VF39 #54 EPS after heating and cooling sample.
