# Supplementary material for: Enhanced Rheological and Structural Properties of the Exopolysaccharide from Rhizobium leguminosarum VF39 Through NTG Mutagenesis
Source: Polymers (Basel). 2024 Nov 15;16(22):3179. doi: 10.3390/polym16223179 (PMC11598398; doi:10.3390/polym16223179)
Supplement: Supplementary file 1 [file polymers-16-03179-s001.zip › polymers-3262337-supplementary.pdf]

Enhanced Rheological and Structural Properties of Exopolysaccharide from *Rhizobium leguminosarum* VF39 through NTG Mutagenesis

Kyungho Kim [1,†], Eunkyung Oh[1,†], Sohyun Park[1], Jae-pil Jeong[1], Sobin Jeon[1], Sujin Lee[1], Younghyun Shin[1] and Seunho Jung [1,2*]

1 Department of Bioscience and Biotechnology, Microbial Carbohydrate Resource Bank (MCRB), Konkuk University, 120 Neungdong-ro, Gwangjin-gu, Seoul 05029, Korea

2 Department of System Biotechnology, Microbial Carbohydrate Resource Bank (MCRB), Konkuk University, 120 Neungdong-ro, Gwangjin-gu, Seoul 05029, South Korea

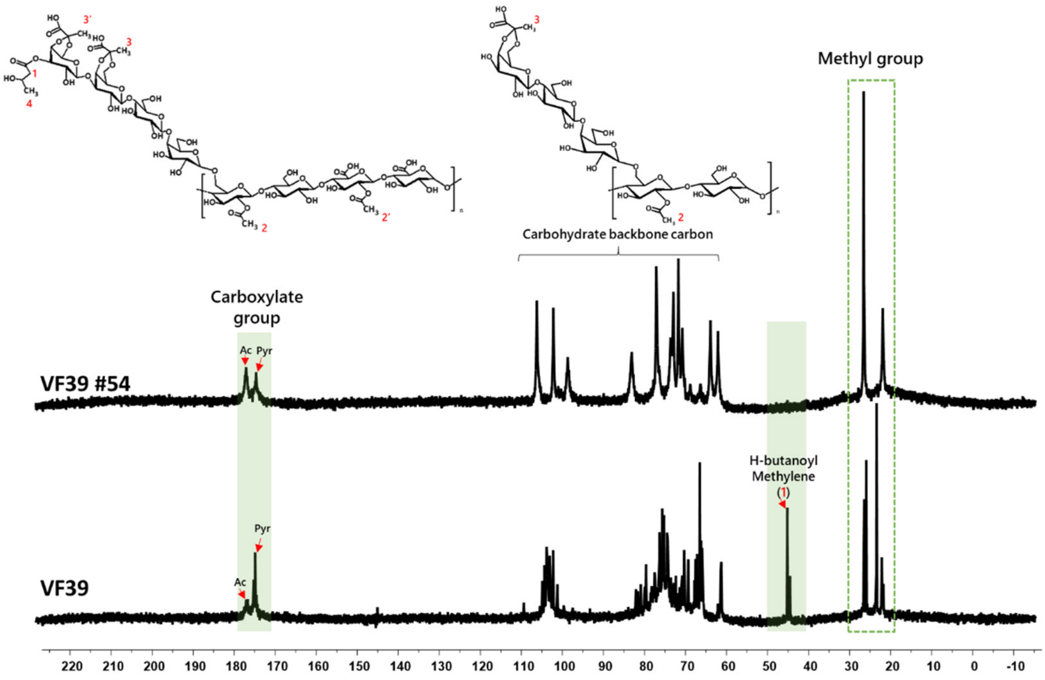

Figure S1. ^{13}C NMR of VF39, and #54 EPS. ^{13}C NMR spectra was measured at 80mg/ml concentration of sample solutions dissolved with D_2O (99%).

The ^{13}C NMR spectrum of VF39 #54 showed the absence of the methylene peak at 45 ppm, which corresponds to the 3-hydroxybutanoyl functional group in the wild-type EPS [1].

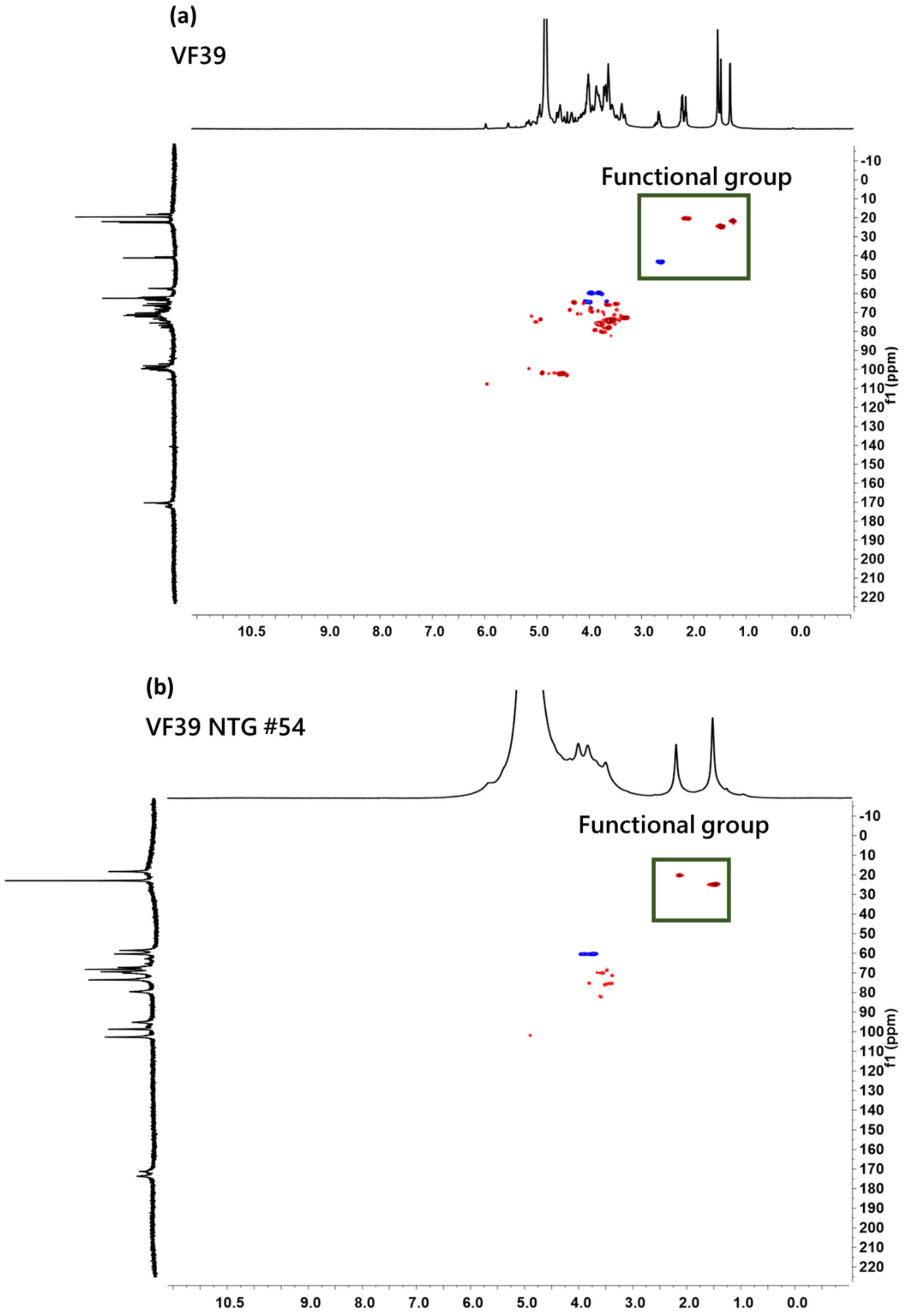

Figure S2. HSQC NMR spectra of VF39, and VF39 #54 EPS. HSQC NMR spectra was measured at 80mg/ml concentration of sample solutions dissolved with D_2O (99%).

In Figure S2, the carbohydrate backbone structure of VF39 #54 EPS exhibited fewer signals compared to the VF39 wild type. This reduction was likely due to a decrease in the number of functional groups and mon-omers that constitute the backbone of VF39 #54 EPS. In this result, no signal for the 3-hydroxybutanoyl functional group was detected in the VF39 #54 EPS [2].

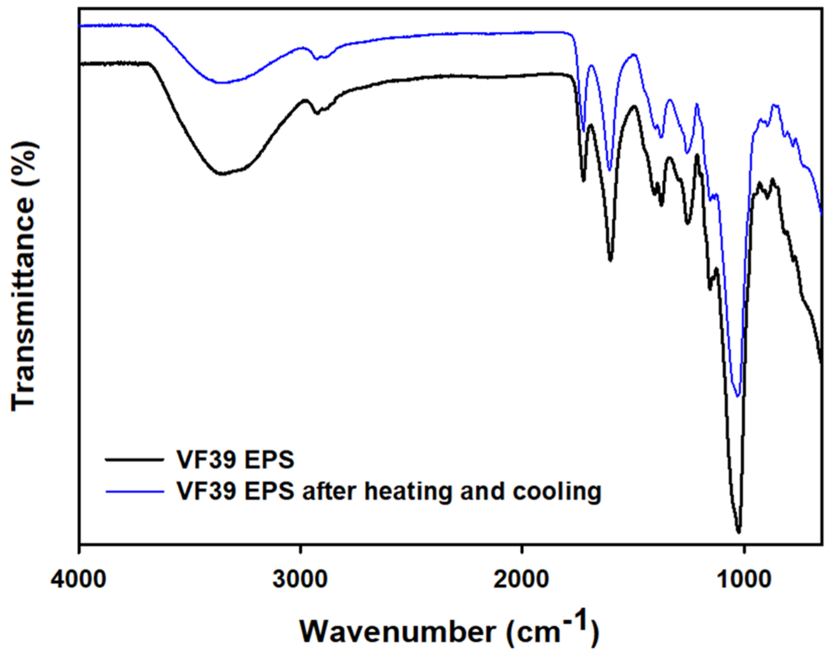

Figure S3. FT-IR spectra of VF39 EPS, and VF39 EPS after heating and cooling sample.

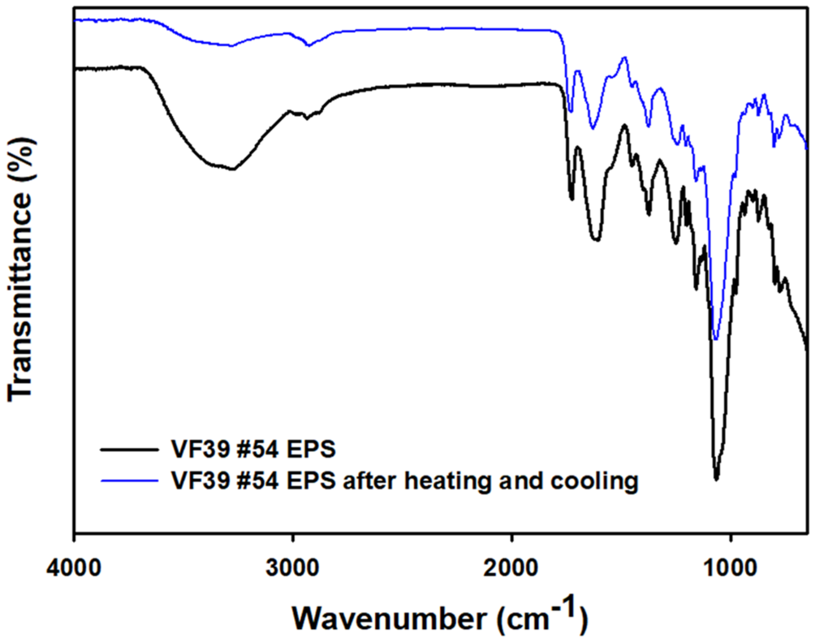

Figure S4. FT-IR spectra of VF39 #54 EPS, and VF39 #54 EPS after heating and cooling sample.

The preservation of the EPS structure after the heating and cooling process was confirmed using FT-IR. EPS sample 2 wt% solution was prepared by heating to 80°C and freeze-drying the cooled sample. In Figures S3 and S4, the FT-IR spectra appeared similar even after the heating process, showing that the structure of the sample was not destroyed.

Reference

[1] **Cremers, H.; Batley, M.; Redmond, J.; Wijfjes, A.; Lugtenberg, B.; Wijffelman, C. Distribution of O-acetyl groups in the exopolysaccharide synthesized by Rhizobium leguminosarum strains is not determined by the Sym plasmid.** *Journal of Biological Chemistry* **1991,** ***266*****, 9556-9564**

[2] **Gao, H.; Yang, L.; Tian, J.; Huang, L.; Huang, D.; Zhang, W.; Xie, F.; Niu, Y.; Jin, M.; Jia, C. Characterization and rheological properties analysis of the succinoglycan produced by a high-yield mutant of Rhizobium radiobacter ATCC 19358.** *International Journal of Biological Macromolecules* **2021,** ***166*****, 61-70.**
